# Identification of genetic variants related to metabolic syndrome by next-generation sequencing

**DOI:** 10.1186/s13098-022-00893-y

**Published:** 2022-08-23

**Authors:** Sanghoo Lee, Seol-A Kim, Jeonghoon Hong, Yejin Kim, Gayeon Hong, SaeYun Baik, Kyeonghwan Choi, Mi-Kyeong Lee, Kyoung-Ryul Lee

**Affiliations:** 1 Center for Companion Biomarker, Seoul Clinical Laboratories Healthcare Inc., 23F, Bldg. A, Heungdeok IT Valley, 13 Heungdeok 1-ro, Giheung-gu, Yongin, Gyeonggi-do 16954 Korea; 2 Central Laboratory, Seoul Clinical Laboratories Healthcare Inc., 23F, Bldg. A, Heungdeok IT Valley, 13 Heungdeok 1-ro, Giheung-gu, Yongin, Gyeonggi-do 16954 Korea; 3 HANARO Medical Foundation, 5F, 1 TOWER, GRAN SEOUL, 33 Jong-ro, Jongno-gu, Seoul, 03159 Korea; 4 Department of MyGenome, Seoul Clinical Laboratories, 28F, Bldg. A, Heungdeok IT Valley, 13 Heungdeok 1-ro, Giheung-gu, Yongin, Gyeonggi-do 16954 Korea

**Keywords:** Metabolic syndrome, Clinical features, Genetic variants, Next-generation sequencing

## Abstract

**Background:**

Metabolic syndrome (MetS) is a cluster of conditions associated with glucose intolerance, hypertension, abdominal obesity, dyslipidemia, and insulin resistance that increase the risk of cardiovascular diseases (CVD) and type 2 diabetes (T2D). Since MetS is known as a complex symptom with a high incidence of genetic factors, it is important to identify genetic variants for each clinical characteristic of MetS.

**Methods:**

We performed targeted next-generation sequencing (NGS) to identify genetic variants related to obesity, blood glucose, triacylglycerol (TG), and high-density lipoprotein (HDL)-cholesterol level, and hypertension in 48 subjects with MetS and in 48 healthy subjects.

**Results:**

NGS analysis revealed that 26 of 48 subjects (54.2%) with MetS had putative non-synonymous variants related to the clinical features of MetS. Of the subjects with MetS, 8 (16.7%) had variants in 4 genes (*COL6A2, FTO, SPARC,* and *MTHFR*) related to central obesity, 17 (35.4%) had variants in 6 genes (*APOB, SLC2A2, LPA, ABCG5, ABCG8,* and *GCKR*) related to hyperglycemia, 3 (6.3%) had variants in 4 genes (*APOA1, APOC2, APOA4,* and *LMF1*) related to hypertriglyceridemia, 8 (16.7%) had variants in 4 genes (*ABCA1, CETP, SCARB1*, and *LDLR*) related to low HDL-cholesterolemia, and 5 (10.4%) had variants in *ADD1* related to hypertension.

**Conclusions:**

Our findings may contribute to broadening the genetic spectrum of risk variants related to the development of MetS.

**Supplementary Information:**

The online version contains supplementary material available at 10.1186/s13098-022-00893-y.

## Introduction

MetS, known as syndrome X, Deadly quartet, or insulin resistance syndrome, is defined by a cluster of five risk factors that increase the likelihood of developing CVD, stroke, and T2D [1]. The 5 risk factors for MetS include hypertension, obesity, hypertriglyceridemia, hyperlipidemia, and hyperglycemia. MetS is diagnosed when someone has three or more of these risk factors. There are also other factors that are likely increase the risk for MetS. These include age, genetic susceptibility, and not getting enough exercise. MetS is known to have different prevalence depending on geographic, racial, and ethnic origins, where its prevalence is estimated to be about 35% in North America [2], 11–26% in Europe [3], and 12–37% in Asia–Pacific region [4].

These clinical features of MetS are also caused by genetic factors, and up to 50% of all MetS cases are reported to be inherited [5]. However, as MetS includes multiple combinations of the effects of more than 3 risk factors, pinpointing a causative genotype is difficult and most MetS cases are considered polygenic. Therefore, inheritance of a single specific risk variant may be less important than the additive effects of many alleles, and thus, determination of multiple risk variants is necessary. Considerable progress has been made in the identification of causes that influence the development of MetS. Especially, population-specific genetic risk factors may help early diagnosis of individuals with high susceptibility to MetS.

In addition, as the major important pathogenic single nucleotide polymorphisms (SNPs) for specific target diseases vary among different populations, it is important to explore the genetic variants for clinical factors of MetS in specific populations.

Currently, comprehensive multiplex genomic sequencing technology approaches including NGS, are accelerating the identification of new molecular biomarker targets [6]. Advances in NGS have provided an unprecedented opportunity for identifying rare variants with moderate-to-large effects. These have increased our understanding of the molecular mechanisms underlying many inherited diseases in outlier populations. Targeted NGS is useful for rapidly identifying common and rare genetic variations [7]. Therefore, it can be an interesting and important work to identify rare variants affecting the development of MetS in specific populations with a single NGS panel in which genes identified for individual factors related to the clinical features of MetS are integrated.

In this study, we aimed to identify relevant genetic variants according to the clinical status of MetS using NGS.

## Materials and methods

### Study population, diagnosis of MetS, and ethical approval

Forty-eight participants were classified into the MetS group through clinical diagnoses based on the results of basic blood tests and health examinations at Health Checkup Center of HANARO Medical Foundation, Seoul, Korea, and 48 participants with no clinical features of MetS were included as the healthy control group (Table 1).The 48 subjects with MetS were diagnosed and enrolled according to a harmonized definition of International Diabetes Federation/National Heart, Lung, and Blood Institute/American Heart Association/International Association for the Study of Obesity [8]. All the subjects with MetS had at least three clinical features among hypertension (≥ 130 mmHg systolic and/or ≥ 85 mmHg diastolic), hyperglycemia (fasting glucose: ≥ 100 mg/dl), elevated TG (≥ 150 mg/dl), decreased HDL-cholesterol (< 40 mg/dl in men; < 50 mg/dl in women), and central obesity (waist circumference > 90 cm in men; > 85 cm in women), which followed a definition of the Korean Academy of Family Medicine [9]. Participants with two or more combination medications, chemotherapy, or anticancer drugs on special medications were excluded from the study. Written informed consent was obtained from all study participants. The study protocol was approved by the Institutional Review Board of Seoul Clinical Laboratory (2018-31-02F).

**Table 1:** Clinical characteristics of study participants

Characteristics	MetS	Control	*P* value
Number of participants	48	48	
Age			
Mean ± SD	48.1 ± 10.9	40.1 ± 11.2	
Range	26–71	26–74	
Clinical status (Mean ± SD)			
Waist circumference (cm)	94.3 ± 8.7	75.7 ± 7.1	< 0.0001
Total cholesterol (mg/dL)	201.5 ± 38.6	191.4 ± 30.5	< 0.001
Triglyceride (mg/dL)	199.9 ± 89.5	82.2 ± 28.1	< 0.0001
HDL-cholesterol (mg/dL)	48.0 ± 11.9	70.6 ± 12.4	< 0.0001
LDL-cholesterol (mg/dL)	116.4 ± 34.7	105.2 ± 35.2	0.1216
Systolic pressure (mmHg)	127.9 ± 9.4	111.7 ± 8.7	< 0.0001
Diastolic pressure (mmHg)	77.9 ± 8.2	66.4 ± 6.2	< 0.0001
Fasting blood glucose (mg/dL)	107.3 ± 13.4	91.0 ± 5.6	< 0.0001
AST (U/L)	32.2 ± 13.2	20.0 ± 5.5	< 0.0001
ALT (U/L)	42.8 ± 28.1	16.4 ± 9.0	< 0.0001
γ-GTP (U/L)	57.2 ± 57.1	17.3 ± 8.6	< 0.0001
Serum creatinine (mg/dL)	0.97 ± 0.2	0.78 ± 0.17	< 0.0001
Insulin (μU/mL)	12.0 ± 6.7	4.7 ± 2.4	< 0.0001

### DNA preparation and selection of target NGS panel genes

Whole blood from all participants was collected and DNA was extracted using MagNa Pure 96 System (Roche Life Science, USA). NGS analysis was performed with NextSeq550 NGS system (Illumina, USA). NGS data were obtained with custom made NGS panel (Agilent Technologies, USA) on 28 selected genes related to the 5 clinical features for target capture sequencing. Gene selection was based on a review of research literature related to the 5 clinical features (central obesity, hyperglycemia, hypertriglyceridemia, low HDL-cholesterolemia, and hypertension) used for diagnosing MetS. A list of target genes that were sequenced is shown in Table 2.

**Table 2:** List of target sequenced genes

Hypertriglyceridemia	Central obesity	Low HDL cholesterolemia	Hyperglycemia	Hypertension
*APOA1*	*COL6A2*	*CETP*	*LPA*	*ADD1*
*APOA4*	*CAV1*	*SCARB1*	*SLC2A2*	*ADM*
*APOA5*	*LEP*	*ABCA1*	*ADIPOQ*	*ADRB2*
*APOC2*	*FTO*	*LDLR*	*APOB*	
*APOC3*	*IGF1*	*LPL*		
*ZHX3*	*SPARC*			
*GPIHBP1*	*MTHFR*			
*LMF1*	*GCKR*			

### Target capture and NGS

DNA extracted from whole blood of study participants was sheared into approximately 180 bp fragments with QSonica Sonicator (QSonica, USA). Sheared DNA was purified with AMPure XP beads (Beckman Coulter, USA) and the NGS library was prepared to target hybridization capture with the NGS panel using an Agilent SureSelectXT Custom Panel with SureSelectXT reagent kit (Agilent Technologies, USA) following the manufacturers’ instructions. Target capture libraries were sequenced on the NextSeq550 platform (Illumina, USA) using 2 × 150 bp paired-end runs.

### Data analyses

All sequenced reads were aligned to the human reference genome National Center for Biotechnology Information build 37 (GRCh37/hg19) using the Burrows-Wheeler Aligner (Ver. 0.7.12). Local re-alignment around the indels and pair-end fixing was performed using Genome Analysis Tool Kit (GATK) Lite (Ver. 2.3-9), and PCR duplicates were removed using Picard (Ver. 1.128). The GATK Unified Genotyper was used to call the genomic variants. Mutated loci were annotated using snpEff (Ver. 4.3q). Non-synonymous single nucleotide variants (SNVs) and indels in the coding exons and splicing sites of target genes were included in the analysis. Known SNP with minor allelic frequency > 5% in the 1000 Genome Project Phase I East Asian (April 2012) and Genome Aggregation Database (gnomAD) (v2.1.1 release) were annotated and removed as the common non-disease-associated SNPs. Variant allele frequency ≤ 35%, total read depth ≤ 10X, and reads supporting a variant allele count ≤ 3 were also rejected as non-significant variants. False-positive indels were removed manually using the Integrated Genome Viewer (IGV Ver. 1.8.0.). In addition, all variants found in the 48 control subjects were rejected as non-disease-associated SNPs. After removing non-significant variants, in silico prediction was performed using SIFT, PolyPhen-2, PROVEAN, and MutationTaster.

According to American College of Medical Genetics (ACMG) guideline, variants identified in this study were classified as pathogenic, likely pathogenic, variant of uncertain significance (VUS), likely benign, and benign. Predicted benign or likely benign variants in more than half of the programs were regarded as benign or likely benign variants and were not regarded as significant variants associated with MetS.

## Results

### Target region of NGS panel and targeted sequencing depth and coverage of each subject

Target capture genomic regions of interest included 359 exons and 109,173 bases. As shown in Additional file 1: Table S1, NGS probes were designed to capture exons in target genes and were used for target capture sequencing on subjects with MetS. The calculated mean depth and on-target ratio were 1099X and 30.8%, respectively. The coverage in the target region was 96.5%, with an average of ≥ 20X non-duplicated reads.

### Variant spectrum from target capture sequencing

**Fig.1:**
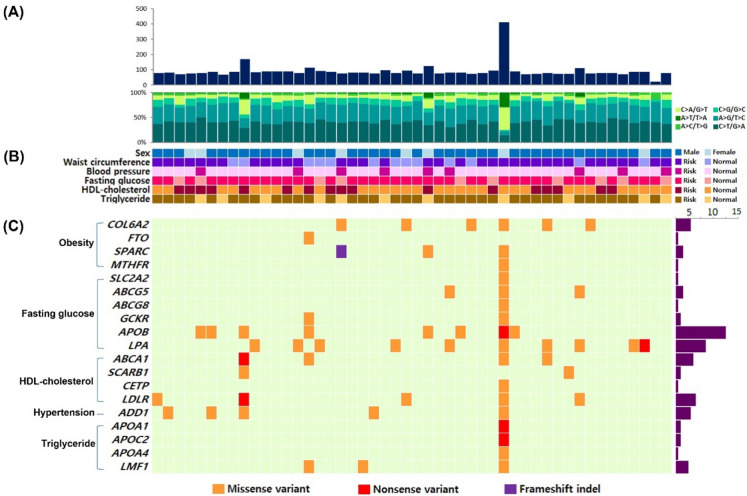
Variant spectrum from target capture sequencing: **A** Overall variant profiles. **B** Characteristics of MetS subjects. Normal and risk subjects were classified according to the definition of MetS. **C** Genes with putative variants (East Asian frequency < 5%) associated with clinical feature of MetS and the number of subjects with following genetic variants (rightmost graph). Each block shown in Fig. 1 represents each individual participant with MetS

After variant calling, synonymous and off-target variants, and those that did not reach the cut-off value were rejected. Variants classified as benign and likely benign by in silico prediction tools were also rejected. We then identified non-synonymous variants associated with the clinical features of MetS, including 74 missense, 9 nonsense, and 1 frameshift indel after common variant removal based on the 1000 Genomes Project (East Asian frequency ≥ 5%) and GenomAD (East Asian frequency ≥ 5%) and compared with the control group data (Table 3). By grouping SNVs according to the nature of the nucleotide changes, we observed enrichment of C:G → T:A transition variants (40.3% on average) followed by A:T → G:C transition variants (33.1% on average) (Fig. 1A). Clinical information for each sample according to variant spectrum is described in Fig. 1B with subjects classified as normal and risk group. Validated loci were further analyzed for the functional prediction of amino acid changes using 4 different prediction algorithms (SIFT, PolyPhen-2, PROVEAN, and Mutation Taster). Overall, 26 of 48 subjects (54.2%) had putative non-synonymous variants associated with the clinical features of MetS (Fig. 1C).

**Table 3:** Genetic variants identified from subjects with MetS

Gene	chr	pos	ref	alt	rs	cDNA change	AA change^a^	1000G_ all (%)^b^	1000G_ EAS (%)^c^	gnomAD_ all (%)^d^	gnomAD_ EAS (%)^e^	Consequence on protein	Pathogenicity (ACMG)	Novelty
*LDLR*	19	11217315	C	T	rs200990725	c.769C > T	p.(Arg257Trp)	0.05		0.01	0.1	missense	Pathogenic	
*LDLR*	19	11227594	G	A	rs201971888	c.1765G > A	p.(Asp589Asn)	0.03	0.13	0.01	0.11	missense	Pathogenic	
*ADD1*	4	2877658	C	T	rs2295497	c.16C > T	p.(Arg6Cys)	0.26	1.29	0.11	1.45	missense	VUS	
*APOB*	2	21227275	C	T	rs772544842	c.11953G > A	p.(Asp3985Asn)	0.05	–	0.01	0.01	missense	VUS	
*APOB*	2	21237462	T	C	rs760835338	c.3700A > G	p.(Met1234Val)	0.03	0.13	< 0.01	0.01	missense	VUS	
*ADD1*	4	2877658	C	T	rs2295497	c.16C > T	p.(Arg6Cys)	0.26	1.29	0.11	1.45	missense	VUS	
*APOB*	2	21224769	A	T		c.13525T > A	p.(Tyr4509Asn)	–	–	–	–	missense	VUS	Novel
*APOB*	2	21225239	A	T		c.13055T > A	p.(Leu4352Gln)	–	–	–	–	missense	VUS	Novel
*APOB*	2	21232602	C	A		c.7138G > T	p.(Val2380Phe)	0.03	–	–	–	missense	VUS	Novel
*APOB*	2	21233085	G	A	rs141641980	c.6655C > T	p.(Arg2219Cys)	–	–	0.01	0.01	missense	Likely Pathogenic	
*APOB*	2	21233202	G	T		c.6538C > A	p.(Gln2180Lys)	–	–	–	–	missense	VUS	Novel
*APOB*	2	21233260	T	A		c.6480A > T	p.(Leu2160Phe)	–	–	–	–	missense	VUS	Novel
*APOB*	2	21260063	G	A	rs886055594	c.602C > T	p.(Thr201Ile)	0.03	0.13	–	–	missense	VUS	
*ADD1*	4	2877658	C	T	rs2295497	c.16C > T	p.(Arg6Cys)	0.26	1.29	0.11	1.45	missense	VUS	
*ABCA1*	9	107547916	C	A		c.6406G > T	p.(Gly2136Ter)	0.03	–	–	–	nonsense	Pathogenic	Novel
*ABCA1*	9	107549257	C	A		c.6205G > T	p.(Asp2069Tyr)	0.03	–	–	–	missense	Pathogenic	Novel
*ABCA1*	9	107583676	C	A		c.2940G > T	p.(Gln980His)	0.03	–	–	–	missense	Likely Pathogenic	Novel
*SCARB1*	12	125294817	C	A		c.745G > T	p.(Asp249Tyr)	0.03	–	< 0.01	–	missense	Likely Pathogenic	Novel
*LDLR*	19	11240340	C	A		c.2541C > A	p.(Tyr847Ter)	0.03	–	–	–	nonsense	Pathogenic	Novel
*LPA*	6	161015089	C	T	rs758209955	c.3530G > A	p.(Arg1177Gln)	0.05	–	< 0.01	0.03	missense	VUS	
*LPA*	6	161027656	G	C	rs373258692	c.2638C > G	p.(Pro880Ala)	0.08	–	0.01	0.02	missense	VUS	
*APOB*	2	21246542	C	A		c.2459G > T	p.(Gly820Val)	0.03	–	–	–	missense	VUS	Novel
*GCKR*	2	27720461	G	A	rs1296302285	c.249G > A	p.(Met83Ile)	0.03	0.13	< 0.01	< 0.01	missense	Pathogenic	
*ABCA1*	9	107560828	C	A		c.4995G > T	p.(Met1665Ile)	0.03	–	–	–	missense	Likely Pathogenic	Novel
*ABCA1*	9	107593987	C	T		c.1631G > A	p.(Gly544Asp)	0.05	–	–	–	missense	Likely Pathogenic	Novel
*LMF1*	16	904592	G	T		c.1644C > A	p.(Ser548Arg)	0.03	–	–	–	missense	VUS	Novel
*FTO*	16	53860052	G	A	rs79206939	c.400G > A	p.(Ala134Thr)	0.44	2.18	0.21	2.77	missense	VUS	
*LPA*	6	160953627	T	C	rs759203171	c.5897A > G	p.(Glu1966Gly)	0.03	0.13	< 0.01	0.07	missense	Likely Pathogenic	
*SPARC*	5	151043016	CTA	C	rs71757813	c.1024_1025delTA	p.(Ter342fs)	1.10	0.1	1.39	0.06	frameshift & stop lost	Pathogenic	
*COL6A2*	21	47552333	T	C	rs200200671	c.2927T > C	p.(Leu976Ser)	0.03	0.13	0.02	0.12	missense	VUS	
*LMF1*	16	904615	C	T	rs377058908	c.1621G > A	p.(Gly541Arg)	0.05	–	0.02	0.02	missense	Pathogenic	
*ADD1*	4	2877658	C	T	rs2295497	c.16C > T	p.(Arg6Cys)	0.26	1.29	0.11	1.45	missense	VUS	
*LPA*	6	160961120	A	G		c.5690T > C	p.(Phe1897Ser)	0.03	–	–	–	missense	Likely Pathogenic	Novel
*LDLR*	19	11213345	G	T		c.196G > T	p.(Val66Phe)	0.03	–	–	–	missense	VUS	Novel
*COL6A2*	21	47552350	A	G	rs190664941	c.2944A > G	p.(Met982Val)	0.24	1.09	0.11	1.28	missense	VUS	
*APOB*	2	21231474	C	A		c.8266G > T	p.(Gly2756Cys)	0.03	–	–	–	missense	VUS	
*APOB*	2	21242602	C	A	rs749048977	c.2992G > T	p.(Asp998Tyr)	0.03	–	< 0.01	< 0.01	missense	VUS	
*SPARC*	5	151051211	G	T		c.253C > A	p.(Leu85Met)	0.03	–	–	–	missense	Likely Pathogenic	Novel
*ABCG5*	2	44055127	T	G		c.629A > C	p.(Asp210Ala)	1.96	1.83	–	–	missense	Pathogenic	Novel
*LPA*	6	160968904	A	T		c.5221T > A	p.(Cys1741Ser)	–	–	–	–	missense	VUS	Novel
*APOB*	2	21229457	G	T	rs764056784	c.10283C > A	p.(Thr3428Asn)	0.03	–	< 0.01	0.01	missense	VUS	
*COL6A2*	21	47532049	C	T	rs201842315	c.272C > T	p.(Ala91Val)	0.05	–	< 0.01	0.02	missense	VUS	
*MTHFR*	1	11853996	A	C	rs1357376759	c.1498T > G	p.(Trp500Gly)	–	–	< 0.01	–	missense	Pathogenic	
*APOB*	2	21225441	T	A		c.12853A > T	p.(Lys4285Ter)	–	–	–	–	nonsense	Pathogenic	Novel
*APOB*	2	21225500	A	T		c.12794T > A	p.(Val4265Glu)	–	–	–	–	missense	VUS	Novel
*APOB*	2	21232343	A	T		c.7397T > A	p.(Leu2466Ter)	–	–	–	–	nonsense	Pathogenic	Novel
*APOB*	2	21234331	G	T		c.5409C > A	p.(Tyr1803Ter)	0.03	–	–	–	nonsense	Pathogenic	Novel
*APOB*	2	21239521	C	A		c.3122G > T	p.(Gly1041Val)	0.03	–	–	–	missense	Pathogenic	Novel
*APOB*	2	21245813	G	T	rs1801700	c.2706C > A	p.(Asn902Lys)	–	–	< 0.01	–	missense	VUS	
*GCKR*	2	27720219	G	T		c.169G > T	p.(Asp57Tyr)	0.03	–	–	–	missense	Likely Pathogenic	Novel
*ABCG5*	2	*44064984*	A	T		c.254T > A	p.(Leu85Gln)	–	–	–	–	missense	VUS	Novel
*ABCG8*	2	44079943	G	A	rs140231607	c.900G > A	p.(Met300Ile)	–	–	< 0.01	–	missense	VUS	
*SLC2A2*	3	170715746	G	T		c.1521C > A	p.(His507Gln)	0.03	–	–	–	missense	VUS	Novel
*ADD1*	4	2901004	G	C		c.1003G > C	p.(Ala335Pro)	0.08	–	–	–	missense	Likely Pathogenic	Novel
*ADD1*	4	2901052	G	C		c.1051G > C	p.(Ala351Pro)	0.08	–	–	–	missense	VUS	Novel
*SPARC*	5	151051234	C	A		c.230G > T	p.(Cys77Phe)	0.03	–	–	–	missense	Likely Pathogenic	Novel
*LPA*	6	160952816	T	A		c.6068A > T	p.(Tyr2023Phe)	–	–	–	–	missense	Likely Pathogenic	Novel
*LPA*	6	160952847	C	A		c.6037G > T	p.(Gly2013Cys)	0.03	–	–	–	missense	VUS	Novel
*LPA*	6	160977150	G	T		c.4880C > A	p.(Thr1627Lys)	0.03	–	–	–	missense	VUS	Novel
*ABCA1*	9	107547714	A	T		c.6608T > A	p.(Ile2203Lys)	–	–	–	–	missense	Likely Pathogenic	Novel
*APOA4*	11	116693885	A	T		c.23T > A	p.(Leu8Gln)	–	–	–	–	missense	VUS	Novel
*APOA1*	11	116706664	C	A	rs121912717	c.664G > T	p.(Glu222Ter)	–	–	< 0.01	–	nonsense	Pathogenic	
*APOA1*	11	116707841	C	A		c.76G > T	p.(Glu26Ter)	0.03	–	–	–	nonsense	Likely Pathogenic	Novel
*LMF1*	16	920754	C	A		c.1207G > T	p.(Val403Phe)	0.03	–	–	–	missense	VUS	Novel
*LMF1*	16	920784	T	A		c.1177A > T	p.(Met393Leu)	–	–	–	–	missense	VUS	Novel
*LMF1*	16	943068	A	T		c.668T > A	p.(Leu223Gln)	–	–	–	–	missense	Likely Pathogenic	Novel
*CETP*	16	57003838	G	T		c.452G > T	p.(Gly151Val)	0.03	–	–	–	missense	VUS	Novel
*LDLR*	19	11213346	T	A		c.197T > A	p.(Val66Asp)	–	–	–	–	missense	VUS	Novel
*LDLR*	19	11216153	C	A		c.571C > A	p.(Gln191Lys)	0.03	–	–	–	missense	Likely Pathogenic	Novel
*LDLR*	19	11221432	C	A		c.1045C > A	p.(Gln349Lys)	0.03	–	–	–	missense	Likely Pathogenic	Novel
*APOC2*	19	45452107	G	T	rs148445956	c.205G > T	p.(Glu69Ter)	0.02	–	< 0.01	–	nonsense	Pathogenic	
*APOC2*	19	45452116	A	T		c.214A > T	p.(Arg72Trp)	–	–	–	–	missense & splice region	Pathogenic	Novel
*COL6A2*	21	47549172	G	T		c.2524G > T	p.(Ala842Ser)	0.03	–	–	–	missense	VUS	Novel
*APOB*	2	21235376	A	T	rs756610684	c.4364T > A	p.(Phe1455Tyr)	–	–	< 0.01	0.01	missense	Likely Pathogenic	
*LPA*	6	161010726	G	T	rs765360409	c.3806C > A	p.(Pro1269His)	0.03	–	0.01	0.09	missense	VUS	
*ABCA1*	9	107607775	C	T		c.796G > A	p.(Gly266Arg)	0.05	–	–	–	missense	Likely Pathogenic	Novel
*COL6A2*	21	47539759	G	A		c.1327G > A	p.(Glu443Lys)	0.03	0.13	–	–	missense	VUS	Novel
*SCARB1*	12	125348263	C	T	rs4238001	c.4G > A	p.(Gly2Ser)	6.43	0.3	10.2	0.03	missense	Likely Pathogenic	
*ABCG5*	2	44051362	G	T		c.1114C > A	p.(Leu372Met)	0.26	–	–	–	missense	Likely Pathogenic	Novel
*LPA*	6	161022023	G	A		c.3053C > T	p.(Ser1018Leu)	0.03	0.13	–	–	missense	VUS	Novel
*LDLR*	19	11217360	A	T		c.814A > T	p.(Asn272Tyr)	–	–	–	–	missense	VUS	Novel
*COL6A2*	21	47552350	A	G	rs190664941	c.2944A > G	p.(Met982Val)	0.24	1.09	0.11	1.28	missense	VUS	
*LPA*	6	160953627	T	C	rs759203171	c.5897A > G	p.(Glu1966Gly)	0.03	0.13	< 0.01	0.07	missense	Likely Pathogenic	
*LPA*	6	161006178	G	A	rs200154828	c.4189C > T	p.(Arg1397Ter)	0.03	0.13	< 0.01	0.02	nonsense	Pathogenic	

^a^Amino acid change

^b^Frequency in 1000 Genome Project

^c^Frequency in East Asian from 1000 Genome Project

^d^Frequency in gnomAD

^e^Frequency in East Asian from gnomAD

### Variant related to central obesity

*CAV1*, *COL6A2*, *FTO*, *LEP*, *IGF1*, *SPARC*, *GCKR*, and *MTHFR* genes were included for the NGS analysis for detecting variants related to central obesity. Eleven types of variants in 4 genes (*COL6A2*, *FTO*, *SPARC*, and *MTHFR*) were found to be related to central obesity, and 8 of 48 subjects with MetS (16.7%) showed putative non-synonymous variants in these genes (Fig. 1C). Six missense variants in *COL6A2* were found in 6 subjects with MetS, 1 subject had a missense variant in *FTO*, 3 subjects had 2 missense variants and 1 frameshift indel in *SPARC*, and 1 subject had a missense variant in *MTHFR*.

### Variant related to hyperglycemia

*ADIPOQ*, *APOB*, *SLC2A2*, *LPA*, *ABCG5*, *ABCG8,* and *GCKR* genes were included in the NGS analysis for detecting variants related to fasting blood glucose level. Seventeen of 48 subjects (35.4%) had putative non-synonymous variants in *APOB*, *SLC2A2*, *LPA*, *ABCG5*, *ABCG8,* and *GCKR* (Fig. 1C and Table 3). Eight subjects with MetS had 17 missense variants and 3 nonsense variants in *APOB*. One subject had a missense variant in *SLC2A2*, 3 subjects had 3 missense variants in *ABCG5*, 1 subject had a missense variant in *ABCG8*, 2 subjects had 2 missense variants in *GCKR*, and 10 subjects with MetS had 11 missense variants and 1 nonsense variant in *LPA*.

### Variant related to hypertriglyceridemia

*APOA1*, *APOA4*, *APOA5*, *APOC2*, *APOC3*, *ZHX3*, *GPIHBP1*, and *LMF1* genes were included for the NGS analysis for detecting variants related to hypertriglyceridemia. Overall, 3 of 48 subjects (6.3%) had putative non-synonymous variants in *APOA1*, *APOC2*, *APOA4,* and *LMF1* (Fig. 1C and Table 3). One subject with MetS had 10 non-synonymous variants in *APOA1*, *APOC2*, *APOA4,* and *LMF1*, including 7 missense variants and 3 nonsense variants. The other 2 subjects had 2 missense variants in *LMF1*.

### Variant related to low HDL-cholesterolemia

Among the low HDL-cholesterolemia-related genes (*ABCA1*, *CETP*, *SCARB1*, *LPL*, and *LDLR*) included in the NGS panel, *ABCA1*, *CETP*, *SCARB1*, and *LDLR* showed putative non-synonymous variants in subjects with MetS. Eight of 48 subjects with MetS (16.7%) showed variants in *ABCA1*, *CETP*, *SCARB1*, and *LDLR* (Fig. 1C and Table 3). Four subjects with MetS had non-synonymous variants in *ABCA1* with 6 missense variants and 1 nonsense variant. Two subjects with MetS had 2 missense variants in *SCARB1*. One subject with MetS had a missense variant in *CEPT*, 5 subjects with MetS had 7 missense variants and 1 nonsense variant in *LDLR*.

### Variant related to hypertension

*ADD1*, *ADM*, and *ADRB2* were included for the NGS analysis for detecting variants related to hypertension. Overall, 5 of 48 subjects (10.4%) had putative non-synonymous variants in *ADD1*. Five subjects with MetS had 6 missense variants in *ADD1* (Fig. 1C and Table 3). Four subjects with MetS had the same c.16C > T p.(Arg6Cys) variant and 1 had c.1003G > C p.(Ala335Pro) and c.1051G > C p.(Ala351Pro) variants.

## Discussion

Our NGS analyses identified 84 non-synonymous variants related to the 5 clinical features of MetS in the 19 genes, including 74 missense and 9 nonsense SNPs, and 1 frameshift indel variant. The 8, 17, 3, 8, and 5 subjects with MetS had the 11 variants in 4 genes (*COL6A2*, *FTO*, *SPARC,* and *MTHFR*) related to obesity, 39 in 6 genes (*APOB*, *SLC2A2*, *LPA*, *ABCG5*, *ABCG8*, and *GCKR*) related to hyperglycemia, 10 in 4 genes (*APOA1*, *APOC2*, *APOA4*, and *LMF1*) related to hypertriglyceridemia, 18 in 4 genes (*ABCA1*, *CETP*, *SCARB1*, and *LDLR*) related to low HDL-cholesterolemia, and 6 in 1 gene (*ADD1*) related to hypertension, respectively. To our knowledge, 50 variants identified in our NGS analysis are novel ones that may be related to the clinical features of MetS.

The contribution of genetic factors to the inter-individual variation in obesity accounts for 40–70% [10]. Previous studies have shown that *COL6A2* is a putative preadipocyte marker gene [11] and is also involved in the formation of adipocyte extracellular matrix [12]. Although many genetic loci related to obesity have been reported through genome-wide association studies (GWAS) in large cohorts of European [13] or East Asian populations [14] and in Genetics of Noninsulin dependent Diabetes Mellitus (GENNID) multiethnic family study [15], no studies have reported rare variants in *COL6A2* in subjects with obesity. Notably, the c.2524G > T p.(Ala842Ser) and c.1327G > A p.(Glu443Lys) variants in *COL6A2* were absent in the 1000 Genome and gnomAD databases, indicating they may be novel variants. Based on these results, it is presumed that the 6 variants in *COL6A2* may influence the development of obesity. *FTO*, a well-known gene associated with obesity, has been identified as a major genetic contributor to polygenic obesity in a cohort study of European populations [16, 17]. A large cohort study of Korean populations has also shown that *FTO* SNP rs9939609 is significantly associated with body mass index (BMI), a common measure of obesity [14]. However, we identified *FTO* c400G > A p.(Ala134Thr) variant in only 1 obese subject with MetS, suggesting that this variant may be detected with very low frequency in obese Koreans. A previous study has shown that the *SPARC* gene is associated with human obesity and its expression is increased in adipose tissue of obese individuals [18]. A recent study has revealed that subnetworks of key genes such as *SPARC* play roles in regulating known genes for obesity, CVD, and T2D [19]. However, any variants in *SPARC* related to MetS have not been identified. The 3 variants (c.1024_1025delTA p.(Ter342fs), c253C > A p.(Leu85Met), and c.230G > T p.(Cys77Phe) in *SPARC* related to obesity in our study were classified as pathogenic or likely pathogenic according to the ACMG guidelines, suggesting that these variants may be closely related to obesity. Interestingly, the *SPARC* c.253C > A p.(Leu85Met) and c.230G> T p.(Cys77phe) were identified as previously unreported novel variants, as they were absent in the 2 public databases. The Cys677Thr polymorphism in *MTHFR* has been known to be a significant variant associated with an increased risk of obesity [20]. Although the *MTHFR* c.1498T > G p.(Trp500Gly) variant identified in our study is not Cys677Thr, the variant we identified needs further functional study as it has been classified as pathogenic according to the ACMG guidelines.

A previous study has shown that *APOB* variants are significantly related to higher blood glucose levels in patients with T2D [21]. However, many studies have focused on investigating an association of *APOB* variants with metabolic diseases such as familial hypercholesterolemia [22, 23]. Among the *APOB* variants we identified in this study, c.6655C > T p.(Arg2219Cys), c.12853A > T p.(Lys4285Ter), c.7397T > A p.(Leu2466Ter), c.5409C > A p.(Tyr1803Ter), c.3122G < T p.(Gly1041Val), and c.4364T > A p.(Phe1455Tyr) variants were classified as pathogenic or likely pathogenic according to the ACMG guidelines, suggesting a significant relationship of these variants with higher fasting blood glucose levels in subjects with MetS. Notably, the c.13525T > A p.(Tyr4509Asn), c.13055T > A p.(Leu4352Gln), c.7138G > T p.(Val2380Phe), c.6538C > A p.(Gln2180Lys), c.6480A > T p.(Leu2160Phe), c.2459G > T p.(Gly820Val), c.8266G > T p.(Gly2756Cys), c.12853A > T p.(Lys4285Ter), c.12794T > A p.(Val4265Glu), c.7397T > A p.(Leu2466Ter), c.5409C > A p.(Tyr1803Ter), and c.3122G > T p.(Gly1041Val) in *APOB* were identified as novel variants that had not been previously reported. Thus, further studies are needed to elucidate the functional associations of these novel variants. Two GWA studies have reported that missense variants including the Thr110Ile encoded by rs5400 SNP in *SLC2A2*, known as *GLUT2* are significantly associated with impaired fasting glucose [24, 25]. Interestingly, another study has suggested that study participants with the missense variant, Thr110Ile, in *SLC2A2* show a preference for carbohydrates [26]. However, the missense variant, c1521C > A p.(His507Gln), in the *SLC2A2* related to increased fasting glucose levels that we identified in this study has not been previously reported in GWAS or NGS-based study. Recent cohort studies of MetS in Korean populations have not reported the c1521C > A p.(His507Gln) variant in *SCL2A2* [27, 28]. Therefore, the *SLC2A2* variant we identified may be a novel variant related to hyperglycemia in the Korean population, and additional studies are needed to reveal that the novel variant has a functional association with the phenotypes of MetS. Several studies have also reported that *LPA* variants that are considered risk factors for cardiovascular disease [29] and *GCKR* variants are associated with hyperglycemia [24, 30]. Interestingly, the c.5690T > C p.(Phe1897Ser), c.5221T > A p.(Cys1741Ser), c.6068A > T p.(Tyr2023Phe), c.6037G > T p.(Gly2013Cys), c.4880C > A p.(Thr1627Lys), and c.3053C > T p.(Ser1018Leu) variants in *LPA* and the c.169G > T p.(Asp57Tyr) variants in *GCKR* were absent in the 2 public databases, indicating their novelty. Notably, the c.5897A > G p.(Glu1966Gly), c.5690T > C p.(Phe1897Ser), and c.6068A > T p.(Tyr2023Phe) variants in *LPA* and the c.169G > T p.(Asp57Tyr) variant in *GCKR* were classified as pathogen or likely pathogen according to the ACMG guidelines, suggesting that these novel variants are likely to be significantly related to hyperglycemia in individuals with MetS. In a cohort of patients with T2D, two variants (rs6720173 and rs4148211) in ABC transporter genes, *ABCG5* and *ABCG8*, have been found to increase the risk of T2D in humans [31]. The most significant SNP (rs4299376) in *ABCG8* was also found to be associated with increased fasting plasma glucose levels [32]. In our study, c.629A > C p.(Asp210Ala), c.254T > A p.(Leu85Gln), and c.1114C > A p.(Leu372Met) in *ABCG5* and c.900G > A p.(Met300Ile) in *ABCG8* were found to be related to higher fasting glucose levels. Notably, these 3 variants in *ABCG5* were previously unknown, suggesting that these novel variants may be novel genetic determinants of MetS and be ethnic-specific genetic variants under clinical conditions of Mets.

A GWA study of individuals with hypertriglyceridemia has shown that common variants in 4 genetic loci (*APOB*, *LPL*, *GCKR*, and *APOA5*) are significantly related to increased TG levels [33]. Among genetic variants affecting TG metabolism, a rare *APOC3* p.(Gln38Lys), well-known as a gain-of-function (GOF) variant has been found to contribute to increased TG levels [34]. Our NGS analysis revealed 10 rare variants at 4 genes (*APOA1*, *APOA4*, *APOC2*, and *LMF1*) related to hypertriglyceridemia. Among them, c.76G > T p.(Glu26Ter) in *APOA1*, c.23T > A p.(Leu8Gln) in *APOA4*, c.214A > T p.(Arg72Trp) in *APOC2*, c.1644C > A p.(Ser548Arg), c.1207G > T p.(Val403Phe), c.1177A > T p.(Met393Leu), and c.668T > A p.(Leu223Gln) in *LMF1* were identified as novel variants because these variants were not present in the 2 public databases. Notably, the *APOA1* c.76G > T p.(Glu26Ter), the *APOC2* c.214A > T p.(Arg72Trp), and the *LMF1* c.668T > A p.(Leu223Gln) variants were classified as likely pathogenic, pathogenic, and likely pathogenic according to the ACMG guidelines, respectively, suggesting that these rare variants may be significantly related to hypertriglyceridemia in individuals with MetS.

A previous study has shown that a variant (rs9282541, Arg230Cys) in *ABCA1*, known as ATP-binding cassette transporter A1 gene is significantly associated with low HDL-cholesterolemia in Native Americans, suggesting that the variant is not only exclusive to Native Americans but is also a significant genetic determinant of HDL-cholesterol levels [35]. The 7 variants, c.6406G > T p.(Gly2136Ter), c.6205G > T p.(Asp2069Tyr), c.2940G > T p.(Gln980His), c.4995G > T p.(Met1665Ile), c.1631G > A p.(Gly544Asp), c.6608T > A p.(Ile2203Lys), and c.796G > A p.(Gly266Arg), in *ABCA1* identified in this study were previously unreported. Moreover, these variants were classified as pathogenic or likely pathogenic according to the ACMG guidelines. Given these results, the 7 variants may play roles in the pathogenesis of low HDL-cholesterolemia. Interestingly, we identified 2 nonsense variants in *ABCA1* and *LDLR* related to low HDL-cholesterolemia, c.6406G > T p.(Gly2136Ter) and c.2541C > A p.(Tyr847Ter), respectively. The 2 variants were also classified as pathogenic according to the ACMG guidelines, suggesting that the variants may be significantly related to an increased risk of low HDL-cholesterolemia in individuals with MetS as well as play an important role in the pathogenesis of low HDL-cholesterolemia. Thus, further studies are needed to elucidate the functional associations of these variants.

A previous study has shown that *ADD1* is a salt-sensitive gene that plays a role in the etiology of hypertension [36]. Recently, a study has shown that a genetic polymorphism (rs4961, Gly460Trp) in *ADD1* associated with hypertension is a genetic biomarker of hypertension in Asians [37]. However, the known variant was absent in our study, suggesting that genetic variants affecting the development of MetS may vary across ethnic groups. In our study, since the 4 individuals with MetS had the same c.16C > T p.(Arg6Cys) variant in hypertension-related *ADD1*, this was considered the most frequent variant. Interestingly, the c.1003G > C p.(Ala335Pro) and c.1051G > C p.(Ala351Pro) variants in *ADD* were previously unknown, indicating their novelty.

Our study has several strengths. First, study participants were recruited from the same geographical area, and baseline examinations were conducted at the same medical center using stringent phenotype selection approaches. Second, this study was designed to use subjects with non-medication and no symptoms related to the clinical features of MetS as control subjects. The variant identification process is comparable to that used for high quality data, which greatly enhances the significance of the variant data.

As our study is a DNA-mutation-based cohort study, more accurate results could be obtained by additional in vivo analysis accompanied with RNA or protein expression data. Especially, clinical information and the presence or absence of genetic mutations did not show clearly matched in some of cases, which seem to be because genetic mutations are not necessarily associated with clinical symptoms and not all genes related to clinical symptoms have been screened. Thus, to determine more accurate individual cause genes for each clinical feature, individual cellular protein expression needs to be confirmed to validate the function of mutations, but this was difficult to apply, which is a limitation of the present study.

A recent MetS cohort study involving a total of 8,150 participants revealed that the prevalence of MetS in Koreans over 30 years was about 35.2% [38]. With the increasing incidence of MetS, it is necessary to discover additional genetic variants of MetS. In addition, since the novel variants identified in this study were detected in Koreans with MetS, it is necessary to investigate the potential roles of these variants in other ethnic groups with MetS.

Although common 17 SNPs associated with hypertriglyceridemia and low HDL-cholesterolemia in Korean populations with MetS have recently been reported through GWAS [28], these SNPs are different from the rare non-synonymous variants identified by our NGS analysis. Identifying genetic variants, such as mutations with potential molecular predictors, can enable early identification on risk of MetS and therapeutic targets for drugs. In addition, information regarding genetic factors may help to decide the best decisions on MetS treatment.

## Conclusion

Our study identified 84 non-synonymous variants related to the 5 clinical features of MetS, which include 74 missense and 9 nonsense SNPs, and 1 frameshift indel variant. Among them, 50 variants identified in our NGS analysis are novel ones that may be related to the clinical features of MetS. Our results suggest that the candidate genes and rare non-synonymous variants related to the 5 clinical features of MetS may be used as potential genetic variants or molecular predictors for MetS. However, additional functional studies are needed to validate these novel variants.

## Supplementary Information

**Additional file 1:** **Table S1. **Target region of NGS panel.

## Data Availability

Not applicable.
