# Supplementary material for: Identification of genetic variants related to metabolic syndrome by next-generation sequencing
Source: Diabetol Metab Syndr. 2022 Aug 23;14:119. doi: 10.1186/s13098-022-00893-y (PMC9396768; doi:10.1186/s13098-022-00893-y)

**Supplementary Information**

**Identification of genetic variants related to metabolic syndrome by next-generation sequencing**

Sanghoo Lee^1,*^, Seol-A Kim^1^, Jeonghoon Hong^1^, Yejin Kim^1^, Gayeon Hong^1^, SaeYun Baik^2^, Kyeonghwan Choi^3^, Mi-Kyeong Lee^4^, Kyoung-Ryul Lee^1,2,3,4,*^

^1^Center for Companion Biomarker, Seoul Clinical Laboratories Healthcare Inc., Korea

^2^Central Laboratory, Seoul Clinical Laboratories Healthcare Inc., Korea

^3^HANARO Medical Foundation, Korea

^4^Department of MyGenome, Seoul Clinical Laboratories, Korea

*Corresponding authors:

Sanghoo Lee, Ph.D.

^1^Center for Companion Biomarker, Seoul Clinical Laboratories (SCL) Healthcare Inc., 23F, Bldg. A, Heungdeok IT Valley, 13 Heungdeok 1-ro, Giheung-gu, Yongin, Gyeonggi-do, 16954 Korea

E-mail: [sprout30@scllab.co.kr](mailto:sprout30@scllab.co.kr)

Kyoung-Ryul Lee, M.D., Ph.D.

^1^Center for Companion Biomarker, Seoul Clinical Laboratories Healthcare Inc., 23F, Bldg. A, Heungdeok IT Valley, 13 Heungdeok 1-ro, Giheung-gu, Yongin, Gyeonggi-do, 16954 Korea;

^2^HANARO Medical Foundation, 5F, 1 TOWER, GRAN SEOUL, 33 Jong-ro, Jongno-gu, Seoul, 03159 Korea;

^3^Central Laboratory, Seoul Clinical Laboratories Healthcare Inc., 23F, Bldg. A, Heungdeok IT Valley, 13 Heungdeok 1-ro, Giheung-gu, Yongin, Gyeonggi-do, 16954 Korea;

^4^Department of MyGenome, Seoul Clinical Laboratories, 28F, Bldg. A, Heungdeok IT Valley, 13 Heungdeok 1-ro, Giheung-gu, Yongin, Gyeonggi-do, 16954 Korea

E-mail: dkrlee@scllab.co.kr

**Supplementary Table S1.** Target region of NGS panel.
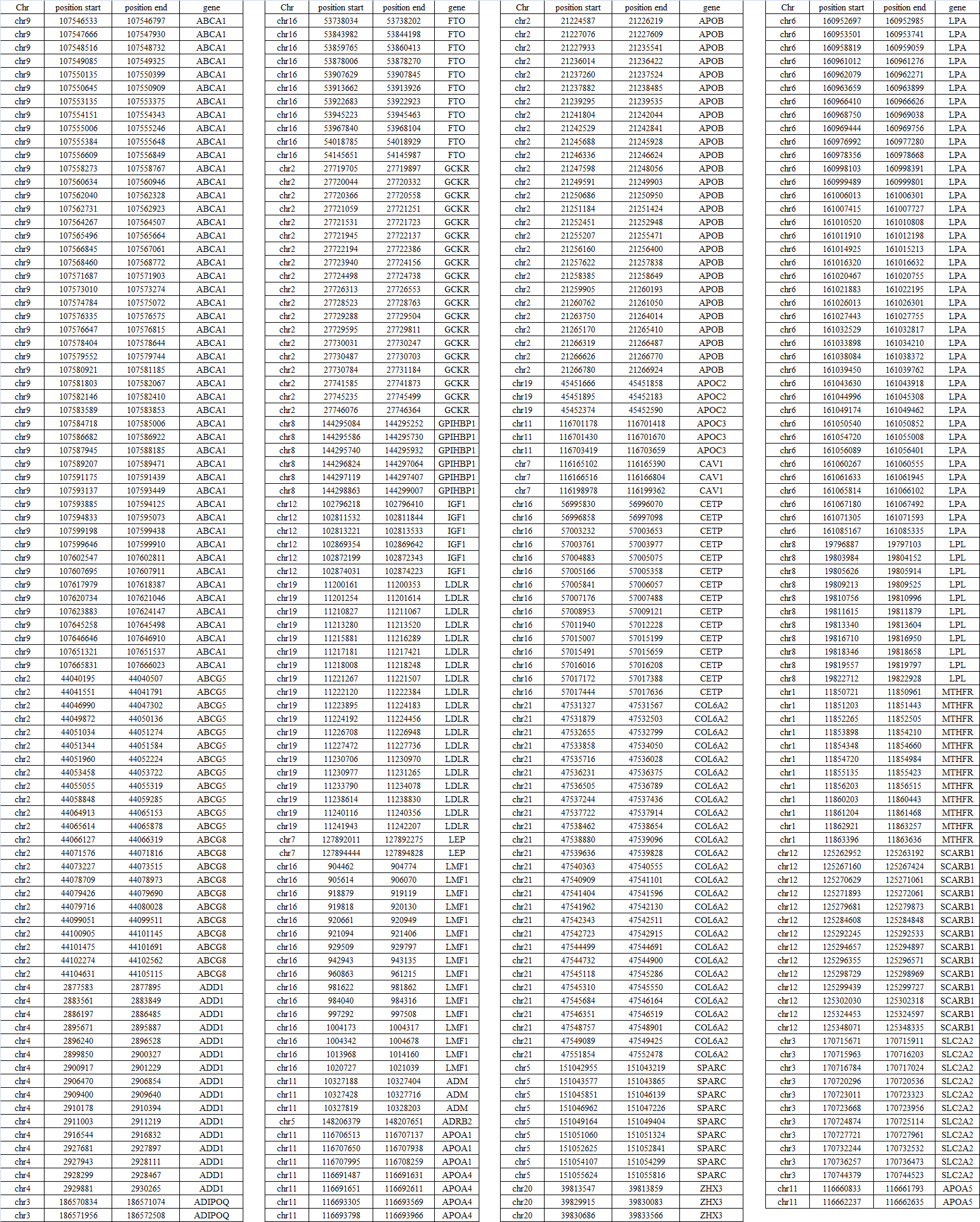

Supplement: Supplementary file 1 — Additional file 1: Table S1. Target region of NGS panel. [file 13098_2022_893_MOESM1_ESM.docx]
